# Effects of global postural re-education on stress and sleep quality in health sciences female students: a randomized controlled trial pilot study

**DOI:** 10.3389/fpsyt.2024.1404544

**Published:** 2024-08-28

**Authors:** Manuel Rodríguez-Aragón, David Varillas-Delgado, Javier Gordo-Herrera, Alba Fernández-Ezequiel, Berta Moreno-Heredero, Noelia Valle

**Affiliations:** ^1^ Faculty of Health Sciences, Universidad Francisco de Vitoria, Madrid, Spain; ^2^ Faculty of Experimental Sciences, Universidad Francisco de Vitoria, Madrid, Spain

**Keywords:** cortisol, sleep quality, students, health sciences, self-treatment

## Abstract

**Objective:**

The purpose of this study was to determine, for the first time, whether the application of a self-management program with global postural re-education (GPR) influences stress and sleep quality in female health science students.

**Methods:**

In this randomized controlled trial pilot study, forty-one female health science students were randomized into a control group (n=21) and an intervention group (n=20). Participants underwent 8 weeks of self-management with and without GPR, after familiarization and therapy training. Outcomes included the State-Trait Anxiety Inventory (STAI) questionnaire and cortisol levels in saliva measured with the “CORTISOL Saliva ELISA SA E-6000” kit. Sleep quality was measured with the Pittsburgh Sleep Quality Index (PSQI) and a Sleep Diary; total sleep time (TST), sleep onset latency (SOL), wakefulness after sleep onset (WASO), sleep efficiency (SE), and perceived sleep quality or satisfaction were assessed using the Likert scale.

**Results:**

After self-treatment with GPR, participants in the intervention group showed lower cortisol levels compared to the control group (p = 0.041). Additionally, the intervention group demonstrated statistically significant improvements in sleep quality according to their PSQI (p = 0.010), STAI (p = 0.043), SOL (p = 0.049), and SE (p = 0.002).

**Conclusion:**

This study shows that self-management through GPR helps reduce stress and improve sleep quality in female health science students.

**Clinical Trial Registration:**

https://clinicaltrials.gov/, identifier NCT05488015.

## 1 Introduction

The proper functioning of physiological processes is essential for the biopsychosocial development of humans. Sleep and stress play a crucial role in maintaining homeostasis (1, 2). In the context of higher education, issues with sleep quality and stress are common and can significantly impact both academic performance and individual health (3, 4).

Sleep, characterized by reduced consciousness and diminished response to external stimuli, is a reversible state marked by muscle relaxation and immobility. It typically involves adopting a stereotyped position (1). Insufficient or poor-quality sleep can lead to a range of physiological, psychological, and behavioral changes (5). ormonal processes, especially those involving the hypothalamus-pituitary-adrenal (HPA) axis and the secretion of corticosteroids like cortisol, play a significant role during stress. Stress itself comprises physiological responses that help in adapting to situations threatening homeostasis, affecting both mental and physical health (6). It is associated with various pathologies, including cardiovascular and coronary diseases, increased susceptibility to infections, and even mortality (7).

Particularly in female undergraduate health science students, higher stress levels have been observed (3, 8). Gender differences in cortisol production and perceived stress responses have been noted, with women often showing more pronounced effects (9). This has been specifically corroborated in academic settings (10, 11). Matud et al. (12) concluded that women generally report higher levels of stress compared to men, with a tendency to experience more emotional and physiological stress responses. Therefore, it is important to consider the menstrual cycle’s phases and their influence on cortisol production (13).

Gender also appears to influence sleep quality. Sleep disorders, along with anxiety and depression, are more prevalent in females and constitute a significant health concern (14, 15). Self-management therapies can be particularly beneficial for this population, as they provide tools to better manage stress and improve sleep quality, which are crucial for academic performance and overall health. A systematic review and meta-analysis by Irwin et al. found that women are more vulnerable to the effects of sleep disturbance, showing a higher inflammatory response to poor sleep quality, which has significant health repercussions (16). Global Postural Re-education (GPR), which utilizes active and evolving postures to stretch muscle chains (17, 18), aims to restore muscle coordination, combining breathing management and proprioceptive stimulation (19). GPR’s effects have been explored in various musculoskeletal pathologies like chronic neck pain (20), urinary incontinence (21, 22), ankylosing spondylitis (23), low back pain (24) and temporomandibular disorders (25). It has also been studied in relation to non-musculoskeletal conditions such as Alzheimer’s (26) and Parkinson´s diseases (27). GPR, as a self-management therapy, holds promise for improving both physical and mental health outcomes by enhancing muscle flexibility, reducing physical tension, and promoting relaxation.

The importance of this study lies in its potential to address a critical gap in the current literature regarding the effectiveness of GPR in managing stress and improving sleep quality among female health science students. Given the high prevalence of stress and sleep issues within this demographic, exploring effective, self-managed interventions like GPR can provide valuable insights and practical applications for improving student well-being and academic performance. GPR has no associated material costs and can be performed anywhere, including at home, which, with proper training and supervision by a physiotherapist, enhances its accessibility and feasibility. Additionally, such self-management strategies have the potential to positively impact healthcare systems by reducing associated costs and burdens.

To our knowledge, no study has yet specifically assessed the impact of GPR intervention on stress and sleep quality among high-level female health science students. The hypothesis of this study is that the application of a self-management program with GPR will significantly reduce stress levels and improve sleep quality in female health science students compared to a control group. Thus, the aim of this study is to determine the effect of a self-management program with GPR on stress and sleep quality in this cohort of female health science students.

## 2 Materials and methods

### 2.1 Study design

A single center, randomized, controlled pilot study (National Clinical Trial identifier NCT05488015) was assessed.

### 2.2 Participants

From August to October 2022, female students from health sciences at Francisco de Vitoria University (including physiotherapy, nursing, medicine, and biomedicine) were invited to participate in the trial. Inclusion criteria were: a) being a female student; b) aged between 18-35 years; and c) enrolled in a health sciences program. The exclusion criteria included: a) working in rotating shifts; b) pregnancy; c) use of non-steroidal anti-inflammatory drugs (NSAIDs); d) experiencing acute or subacute back pain; e) having musculoskeletal or neurological injuries associated with sleep disorders; f) diagnosed sleep disorders (such as sleep apnea or circadian rhythm sleep disorder); g) having tumorous, rheumatological, adrenal, or pituitary diseases; and h) receiving other treatments such as acupuncture that could influence the effects of GPR. The responses to the screening questionnaire of potential participants were evaluated by a medical professional from the Faculty of Medicine at the Universidad Francisco de Vitoria to ensure accurate diagnosis and adherence to the exclusion criteria.

All participants provided written informed consent before their participation. The study protocol was approved by the research ethics committee of Francisco de Vitoria University (UFV 18/2021) and adhered to the principles of the Declaration of Helsinki of 1964, as updated in 2013.

### 2.3 Sample size

Sample size calculation was performed using G*Power 3.4 software (28). An *a priori* calculation indicated the need for a specific number of health sciences female students to achieve statistically significant differences between the intervention and control groups. This calculation aimed for an effect size of a 5.1% reduction in stress (with a statistical power of 80% and a type I error set at 5%), based on a previous study that achieved similar results in an intervention group (29). Consequently, a target sample size of 40 participants was determined.

### 2.4 Randomization

After collecting baseline data, participants were randomly allocated in a 1:1 ratio to either an intervention or a control group using an Excel-generated randomization schedule. Data quality control, management, and protocol compliance were regularly verified by research coordinators. Due to the intervention’s nature, blinding participants, care providers, and outcome assessors was not feasible.

### 2.5 Procedure

**Figure 1:**
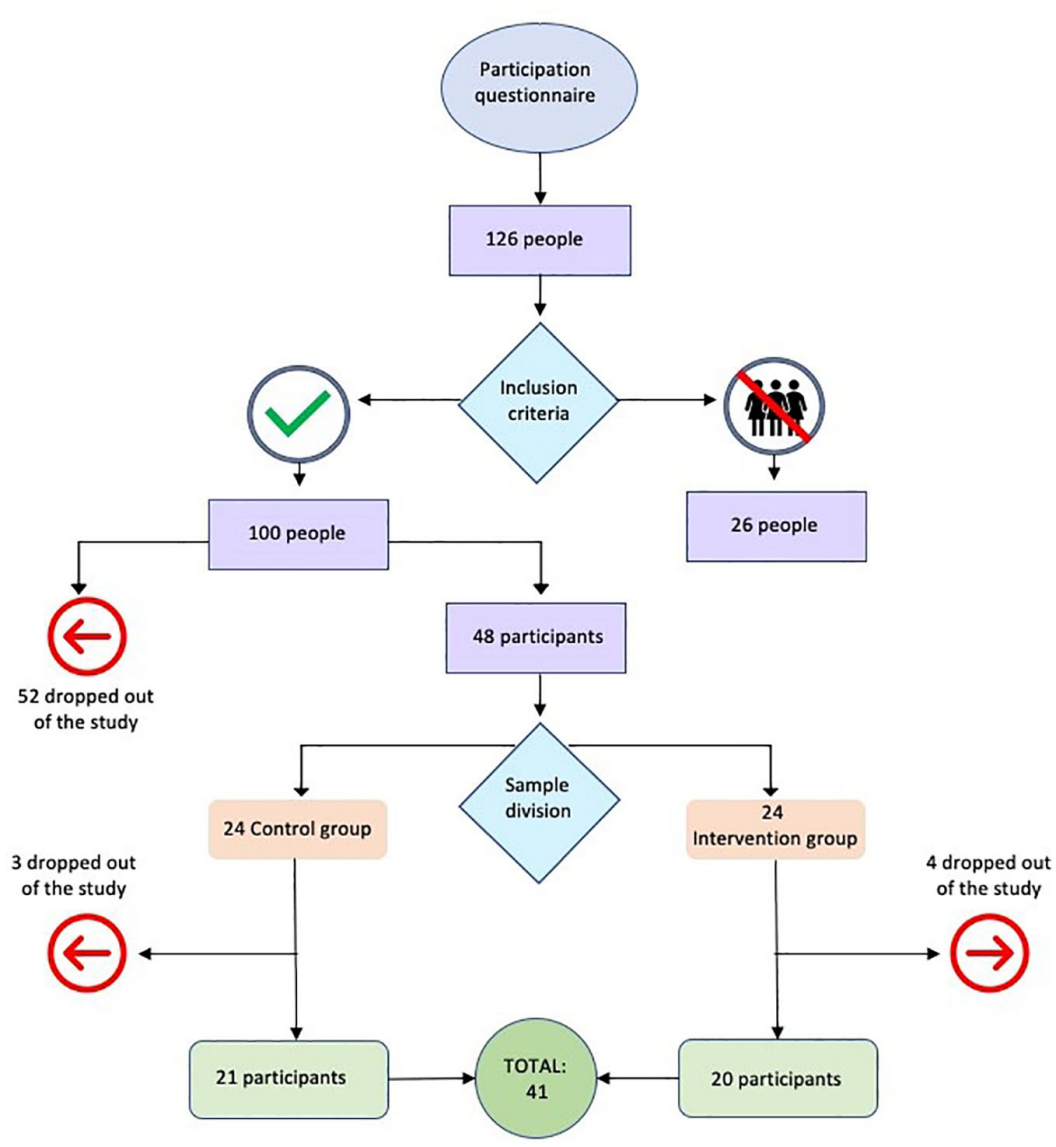
Flow chart.

**Figure 2:**
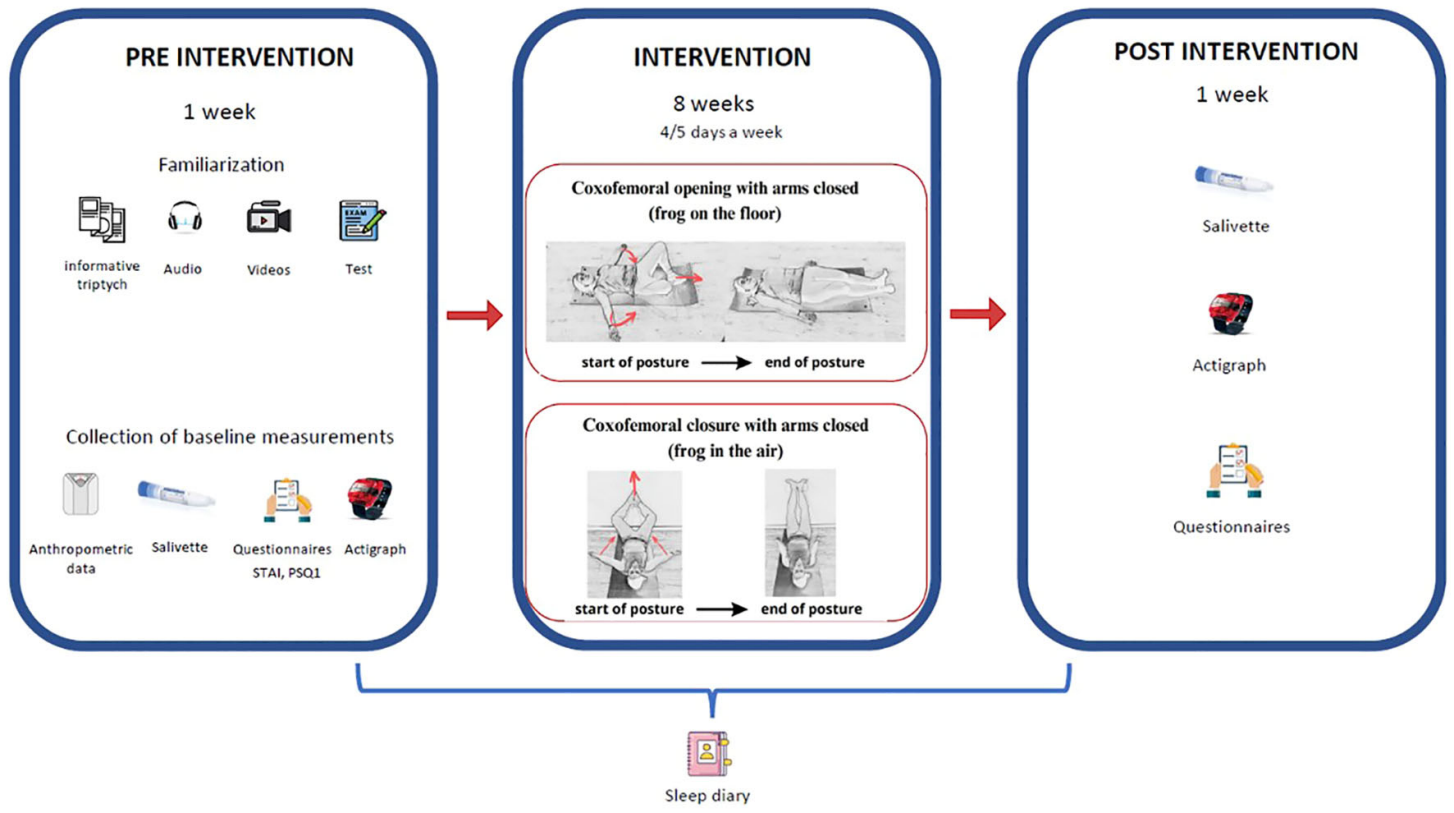
Experimental design.

The experimental group received the following instructions: i) two explanatory videos outlining the postures and their progression throughout the intervention program; ii) a tri-fold brochure detailing each exercise; iii) an audio file providing simultaneous guidance for posture execution; and iv) ongoing researcher support to answer any queries. Participants then completed a questionnaire to evaluate their understanding of the video content. The GPR intervention spanned 8 weeks. Participants were instructed to perform the postures for 4 or 5 days each week, always before bedtime. Two postures were chosen: a coxofemoral opening posture and a coxofemoral closing posture. The coxofemoral opening posture presented hip opening with arms closed (**Figure 1**, intervention): participants had to lie on the floor, with an initial position stabilising the occipital, lumbar and sacral areas, with arms in abduction at 90°, palms facing the ceiling and soles of the feet together and placed close to the gluteal area, thus causing flexion and abduction of the hips and flexion of the knees between 30° and 45°. The position evolved progressively, keeping the occipital, lumbar and sacral areas stabilised, until the arms were closed close to the trunk, with the hips extended, the knees together and in extension, and the feet in a neutral position. In the coxofemoral closing posture, the patients were lying supine, with the hips close to the wall, and the feet resting on the wall. The arms started from 90° of abduction. The occipital and sacrum were stabilised on the floor. The knees open and the soles of the feet together, following the longitudinal axis of the spine. The posture evolved by stretching the legs and bringing them together towards the ceiling, leaning on the wall, maintaining the correct support of the sacral, dorsal and occipital areas, respecting the limits of flexibility of each patient (**Figure 2**). Both postures were performed for 15 minutes for a total treatment time of 30 minutes. The postures selected were unloaded, to facilitate self-management and amplify reactive forces. Breathing, a crucial component of this method, was emphasized, with participants instructed to maintain consistent breathing during posture execution. Additionally, a Sleep Diary was incorporated as a complementary tool alongside the PSQI questionnaire throughout the 8-week intervention.

Breathing, a crucial component of this method, was emphasized, with participants instructed to maintain consistent breathing during posture execution. Additionally, a Sleep Diary was incorporated as a complementary tool alongside the PSQI questionnaire throughout the 8-week intervention.

### 2.6 Primary outcomes measurements

#### 2.6.1 STAI questionnaire

Anxiety, often induced by stress, was measured using the State-Trait Anxiety Inventory (STAI). The STAI assesses trait anxiety (STAI T-A) and state anxiety (STAI S-A), which reflects anxiety levels in response to environmental stimuli. This questionnaire comprises two independent self-assessment scales, each with 20 items rated on a 4-point scale (0 - not at all, 1 - somewhat, 2 - quite a bit, 3 - a lot) (30, 31).

#### 2.6.2 Cortisol levels

Saliva cortisol levels were the primary outcome of this trial. The Salivette Cortisol kit by Sarstedt was utilized for hygienic collection of saliva samples from participants, allowing for subsequent cortisol level measurements as stress indicators. The saliva samples and cortisol concentration were analyzed following the manufacturer’s instructions for the “CORTISOL Saliva ELISA SA E-6000” kit from LDN, Germany.

Participants received both written and audio-visual instructions on correctly collecting saliva samples (Salivette Cortisol, Sarstedt, Spain). Four samples were collected throughout the day to establish cortisol’s circadian rhythm at wake-up, 11:00 am, 3:00 pm, and 20:00. Samples were initially stored at 4°C for up to two days, then preserved at -80°C.

### 2.7 Secondary outcomes measurements

Participants were personally instructed on correctly completing the PSQI questionnaire, Sleep Diary, and menstrual cycle (**Figure 2**).

#### 2.7.1 PSQI

Sleep quality was evaluated using the self-administered PSQI (32). The PSQI is a validated instrument with 19 self-rated items and an additional 5 items rated by a bed partner or roommate. It provides insights into seven sleep aspects: subjective sleep quality, sleep latency, sleep duration, habitual sleep efficiency, sleep disturbances, use of sleep medication, and daytime dysfunction. The first four questions are specifically answered, while the remaining items are scored on a 4-point scale (0-3) (33).

#### 2.7.2 Sleep diary

This self-monitoring tool reports parameters related to sleep quality, such as Total Sleep Time (TST), Sleep Onset Latency (SOL), Wakefulness After Sleep Onset (WASO), Sleep Efficiency (SE), and perceived sleep satisfaction, using a Likert scale ranging from 0 (very poor) to 4 (very good). It includes 9 items asking about 20:00, the moment of attempting to sleep, latency, number and duration of awakenings, time of final awakening, time of getting up, sleep quality, and additional comments (34, 35).

#### 2.7.3 Menstrual cycle

With the menstrual cycle there are variations of different hormones. Likewise, insomnia, insufficient sleep or poor subjective sleep quality are usually more frequent after puberty, pregnancy or menopause, which could be due to hormonal events. It was decided to monitor the menstrual cycle of the participants during the intervention months, in order to estimate the phase of the cycle they were in at the time of applying the questionnaires and collecting saliva (36). The application “My menstrual calendar” was used. The application allowed to enter the exact days of the menstrual period, as well as associated symptoms and sensations.

### 2.8 Statistical analysis

Statistical analyses were performed using Statistical Package for the Social Sciences (SPSS) Statistics for Windows, version 23.0. (IBM Corp., Armonk, USA). Continuous data on cortisol were presented as median (quartile 1 – quartile 3) or, if appropriate, as mean and 95% confidence interval of the mean. Continuous data were given as counts and percentages. Differences in continuous data between groups were assessed with parametric t tests. The time courses of continuous variables were evaluated using two-way Analysis of Variance (ANOVA) with a repeated measurements design (SPSS general linear models), giving readings of a continuous quantity (dependent variable) at two levels of a within-subject factor, and a dichotomous characteristic (e.g., group assignment) as an independent, between-subjects factor. Interactions between the results of a biomarker decline between pre-intervention and post-intervention examinations in the intervention group and control group were analyzed (e.g., results of a biomarker decline between pre-intervention and post-intervention examinations in the intervention group, whereas they stagnated or even rose in the control group). Finally, repeated measurement of variance analysis was conducted to identify potential interaction effects between time and sessions in the study outcomes as follows. To determine whether participants’ anxiety significantly changed, and to uncover potential differences between groups at pre and post assessment, the STAI questionnaire was subjected to statistical analysis. Responses to the State and Trait Anxiety Inventory were scored separately to reveal a state anxiety score and a Trait anxiety score. Menstrual cycle was subject to compare cortisol levels by using one-way ANOVA. The PSQI, TST, WASO, SE and SOL were subject to a two-factor mixed repeated measures ANOVA (2 groups x 2 evaluation times). Significance level was set at p < 0.05.

## 3 Results

Between September and October 2022, two hundred thirty-six female students were assessed for eligibility. One hundred twenty-six applied to participate, and forty-eight, meeting the inclusion criteria, were recruited and randomized into intervention and control groups. Seven participants withdrew from the project without formal explanations. Ultimately, the sample comprised forty-one female students, with twenty-one in the control group and twenty in the intervention group, all of whom completed both pre- and post-intervention assessments (**Figure 1**).

**Table 1** presents detailed baseline data. There were no significant differences between the groups in terms of age, body mass index (BMI), biomarker levels, or outcome scores, ensuring comparability at the outset.

**Table 1:** Baseline characteristics of both the intervention and control group.

	Intervention group	Control group	Effect size	p value
Age years	19.30 (1.16)	19.71 (2.09)	0.057	0.712
Weight, kg	72.76 (7.15)	57.25 (7.39)	-0.55	0.054
Height, cm	165.75 (6.24)	163.67 (6.27)	-0.02	0.296
BMI [kg/m2]	22.84 (2.82)	21.40 (3.46)	-0.143	0.167
Biomarker				
Saliva cortisol [ng/dl]	27.19 (11.63)	30.12 (12.63)	0.403	0.172
Outcomes				
STAI (T-A)	17.9 (6.15)	19.48 (6.83)	0.152	0.59
STAI (S-A)	26.9 (5.50)	25.8 (8.84)	-0.157	0.502
PSQ1	8.75 (1.99)	8.81 (1.72)	0.06	0.645
WASO [min]	7.52 (0.09)	6.01 (0.06)	-0.135	0.637
SE	2.26 (0.50)	2.24 (0.46)	-0.015	0.501
TST [min]	462.15 (61.63)	466.24 (64.93)	-0.048	0.215
SOL	17.22 (8.27)	21.257 (9.33)	0.263	0.128

Data are presented as mean (standard deviation). Continuous data were compared with t tests. Effect sizes for t tests are given as r, for Pearson’s χ² tests as ϕ. P-values below 0.05 were considered statistically significant. BMI, body mass index; STAI (T-A), Trait Anxiety Inventory; STAI (S-A), State Anxiety Inventory; PSQI, Pittsburgh sleep quality index; WASO, wakefulness after the onset of sleep; SE, sleep efficiency; TST, total sleep time; SOL, sleep onset latency at the beginning of night sleep.

**Figure 3:**
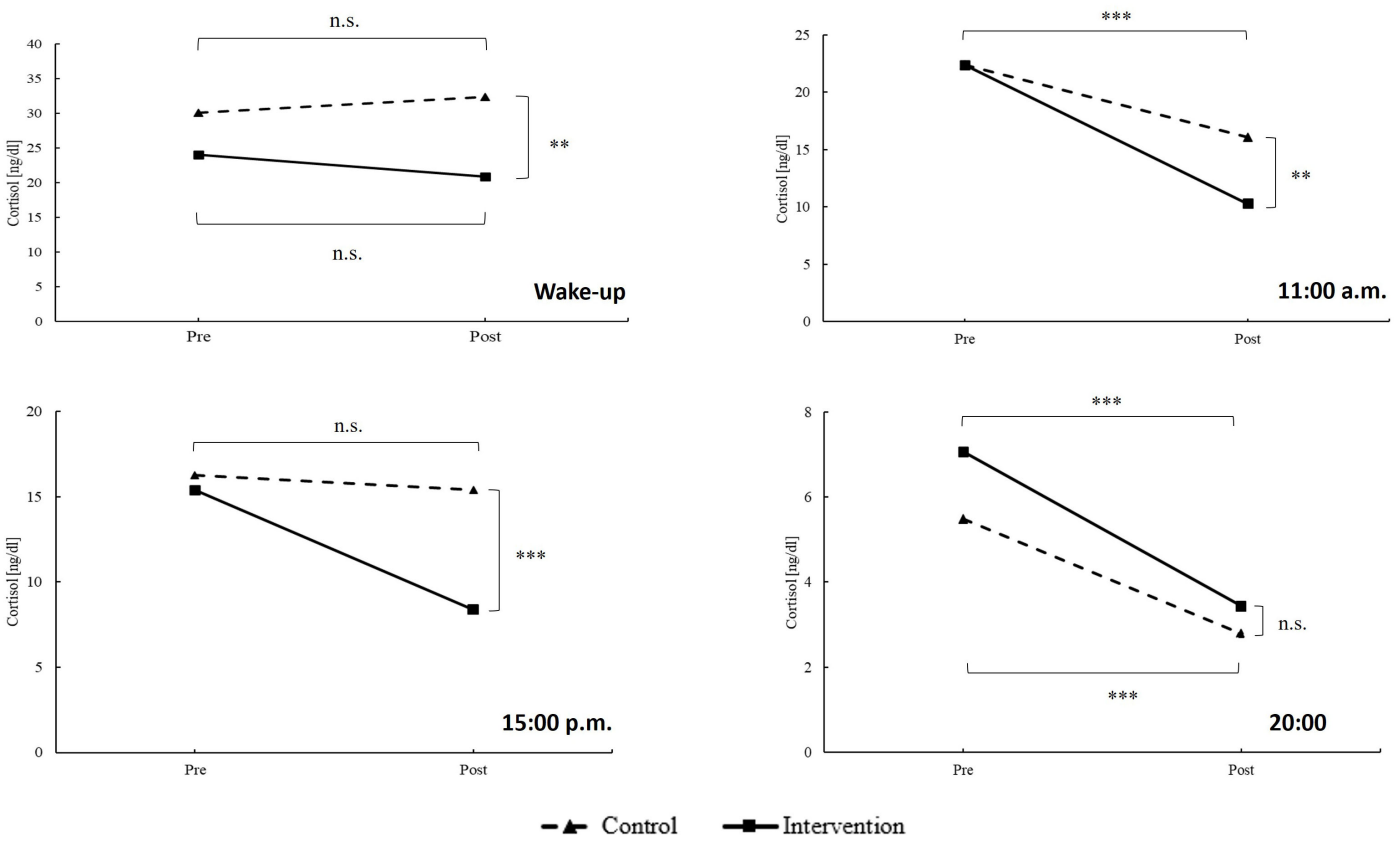
Temporal development of cortisol. Estimated marginal means are calculated by general linear models (two-way ANOVA with repeated measurements design). Data is given as estimated marginal mean. n.s., not significant; **p < 0.010; ***p < 0.001.

**Figure 4:**
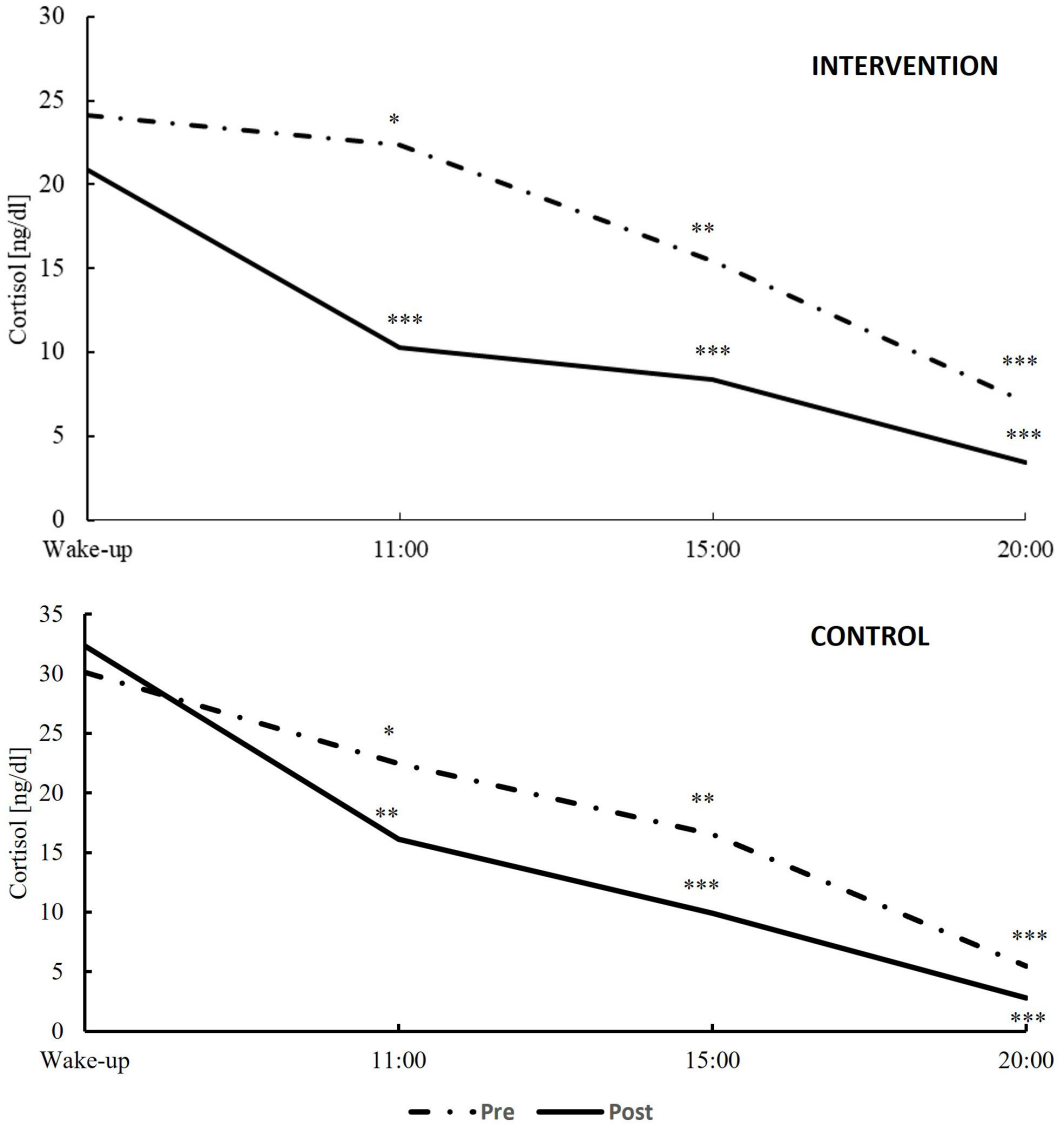
Variation in cortisol day profiles at pre-intervention (dash line) and post-intervention examinations (dark continuous line) within the intervention (top) and the control groups (bottom). *p < 0.05; **p < 0.010; ***p < 0.001.

**Table 2** depicts the changes in cortisol levels from pre- to post-assessment (main effects score), the differences in biomarker levels between intervention and control groups (main effect group), and the variations in biomarker levels over time between the groups (interaction group × biomarker). The temporal trends of these biomarkers are visually represented in **Figures 3**, **4**.

**Table 2:** Differences in cortisol biomarker between pre and post examinations (main effects score), biomarker levels between intervention and control groups (main effect group), and whether temporal developments of biomarker levels vary between groups (interaction group × biomarker).

	Main effect biomarker	Main effect group	Interaction group × biomarker
Cortisol	F = 10.173	F = 55.823	F = 3.094
	df_1_ = 1 df_2_ = 40	df_1_ = 1 df_2_ = 40	df_1_ = 1 df_2_ = 40
	partial η^2^ = 0.441	partial η^2^ = 0.660	partial η^2^ = 0.313
	p = 0.004	p < 0.001	p = 0.041

Effect sizes are given as partial η².

Significant differences were showed in relevant scores from pre- to post-assessment (main effects score), in outcome scores between intervention and control groups (main effect group), and in the temporal progression of scores between groups (interaction group × score), as measured by the State-Trait Anxiety Inventory (STAI) questionnaire and the Sleep Diary. According to the STAI questionnaire, a significant interaction of group x score was observed in the “state” category [STAI-(S-A)] (p = 0.043), where the intervention group showed a greater reduction in scores post-intervention compared to the control group. For the PSQI, both groups significantly lowered their scores at post-intervention (p < 0.001), with the intervention group achieving lower scores than the control group (p = 0.010). WASO, SE, and SOL all demonstrated significant score effects between pre- and post-assessment in both groups (p = 0.008; p = 0.001; and p < 0.001, respectively). Furthermore, for STAI, PSQI, SE, and SOL, statistical differences were observed in the group × score interaction (p = 0.043, p = 0.010, p = 0.002, and p = 0.049 respectively).

Further to these analyses, an exploration into the effects of menstrual cycle phases (preovulation, ovulation, luteal, and menstruation) on cortisol levels and questionnaire outcomes was conducted. This investigation utilized ANOVA to examine the potential differences across these menstrual phases in both pre-intervention and post-intervention measurements. The results indicated no statistically significant changes in cortisol levels across the different menstrual phases, with p-values at wake-up (pre, p = 0.432 and post, p = 0.612), 11:00 (pre, p = 0.056 and post, p = 0.273), 15:00 (pre, p = 0.194 and post, p = 0.393), and 20:00 (pre, p = 0.565 and post, p = 0.639), suggesting that cortisol concentrations were not affected by menstrual cycle phases. Similarly, the analysis of questionnaire outcomes, including the PSQI and STAI, showed no significant differences in scores across menstrual phases, with PSQI values at pre, p = 0.369 and post, p = 0.727, and STAI scores for state (pre, p = 0.893 and post, p = 0.089) and trait (pre, p = 0.890 and post, p = 0.080) anxiety, indicating that the menstrual cycle phases did not significantly influence stress levels or sleep quality among the participants.

## 4 Discussion

This study is the inaugural exploration of a self-management program incorporating GPR and its impact on stress and sleep quality among health sciences students. Over an 8-week period, we analyzed the effects of GPR self-management on university students’ stress and sleep quality. To the best of our knowledge, this is the first study to examine GPR’s influence on these factors in a university student setting.

Our results suggest that GPR treatment positively impacted students. We also evaluated whether this improvement was mirrored in cortisol level reductions throughout the treatment duration. Methodologically, it’s noteworthy to mention the timing of GPR posture execution. While Merinero et al. (12) assert that the benefits of these postures are independent of their execution time, and Kai et al. (37) found enhanced benefits when performed just before sleep. Consistent with these findings, our study supports the benefits of practicing self-management postures before sleep, improving stress management and sleep quality. Self-management and familiarization not only foster greater patient autonomy but could also offer cost savings to the health system and serve as an alternative in times of social distancing. Further studies at various times of the day could solidify these findings.

Participation in this research was completely voluntary, with no compensation in any form, such as academic credits or financial remuneration. The dropout rate could be attributed to a combination of factors: stringent application of exclusion criteria and scheduling conflicts in meeting study requirements. Many participants were juggling academic studies, external practical training in health centers, and employment. This balancing act underscores how varying life conditions, including personal and health factors, significantly influence participant retention rates in research studies (38).

The Cortisol Awakening Response (CAR) is a physiological response in anticipation of the day’s demands. In a situation of chronic stress, cortisol levels remain elevated due to changes in hypothalamic activation of the pituitary. On the other hand, during acute stress, cortisol levels increase, and pulsatility is maintained (39). The study’s results highlighted significant improvements in the group practicing self-management with GPR compared to the control group, with the exception of 20:00, where no differences were observed. Notably, cortisol measurements upon awakening, at 11:00 and at 15:00 showed statistically significant reductions in the intervention group. However, no significant differences in cortisol levels at 20:00 were found between the groups. These results contrast with findings by Carlson et al. (40), who reported no significant changes except at 20:00. Regarding the reduction in CAR, our study could reflect a mitigation of chronic stress, possibly due to improved sleep quality and overall stress reduction induced by GPR. As noted in the review by De Nys et al. (41), the timing of the intervention chosen for this study, before sleep, may promote restorative sleep and thus hormonal balance for cortisol production and regulation. Additionally, the study’s intervention led to a reduction in cortisol levels throughout the day, with the most notable difference at 3:00 pm contributing to a more pronounced stress reduction over the course of the day. These results are supported by the studies of Tortosa-Martínez et al. (42) and Farzane and Koushkie (43), which found a notable reduction in observed cortisol levels during the day following certain physical interventions. The interpretation of the results from the STAI lends consistency to this assertion, reporting a decrease in anxiety values. Lastly, regarding the lack of changes at 20:00, this could be related to the normalization of cortisol levels towards the end of the day, in preparation for sleep (39).

In terms of stress analysis and corroborating the cortisol measurements, the STAI questionnaire provided statistically significant results regarding the participants’ state anxiety (STAI S-A), favoring those who participated in the intervention. However, the trait anxiety component (STAI T-A) remained unchanged. This distinction likely reflects the independence of these two variables (30), suggesting a change in immediate emotional state rather than in the more stable pattern of trait anxiety. This finding aligns with the research of Leal et al. (44), which indicates a lesser correlation between physical issues or threats and the link between trait and state anxiety. It appears that GPR’s emphasis on specific breathing techniques, body awareness, and physical exercise may enhance the initiation of stress coping mechanisms, a benefit also observed in other therapeutic approaches (45).

Regarding sleep quality assessed through the PSQI, significant differences were noted between the intervention and control groups. This indicated an improvement in sleep quality among students who participated in the GPR self-management intervention compared to the control group. According to our review, no previous research has explored sleep quality using the PSQI in the context of a self-management intervention with GPR. However, our findings are consistent with results from other studies where the PSQI was employed as a measurement tool in interventions involving Yoga (46, 47).

The Sleep Diary, utilized for participant support and monitoring, revealed noteworthy results. Statistically significant improvements were observed in sleep efficiency (SE) and latency, while no changes were detected in wakefulness after sleep onset (WASO) and total sleep time (TST) between the intervention and control groups. Similar studies incorporating the Sleep Diary during Yoga interventions (48, 49) reported significant changes in sleep onset latency (SOL), SE, and WASO, aligning with our findings except for WASO. Future research comparing these outcomes with physiological sleep quality measures, such as polysomnography or accelerometers, would be valuable. Sleep plays a critical role in maintaining various physiological functions, including the regulation of neuronal plasticity and synaptic strength essential for memory and cognitive processes (50). Our study suggests that GPR can positively contribute to these sleep-related physiological functions.

Regarding the influence of the menstrual cycle and with results aligned with those of this study, Paludo AC. et al. (51) did not find any influence of the menstrual phase on physiological measures such as emotional state or specific hormonal changes taken after exercise, both aerobic and anaerobic. In any case, it seems of interest to expand research in this direction, due to physiological and psychometric factors that present a great inter-variability in hormonal responses.

Our findings on the significant improvement in sleep quality and stress reduction through GPR among health sciences female students echo results from a preceding study that investigated GPR’s effect on women university lecturers (29). The consistency of outcomes across different university populations suggests the robustness of GPR as an intervention for enhancing sleep quality and managing stress, further emphasizing the need for its integration into university health programs.

Despite the strengths presented in this randomized controlled trial, the study has some potential limitations: a) the self-management that patients perform could be amplified with the manual treatment of the therapist; b) the impossibility of doing face-to-face familiarization sessions. Adding face-to-face sessions and management by the therapist to the familiarization process would be advantageous; c) the fatigue that some participants may have experienced in filling in the Sleep Diary, which could have influenced their natural behavior; d) objective sleep measurement was not conducted, which may affect the accuracy of sleep-related findings; e) the results may not be generalizable to a broader population due to the specific demographic and characteristics of the study sample; f) the diet of participants was not controlled, which could have impacted cortisol levels; g) the specific types of contraceptives used by female participants were not measured, which could affect cortisol response to stress; h) the sample size was relatively small, which may limit the generalizability of the findings. Larger sample sizes are needed in future studies to validate these results and provide more robust conclusions; and i) the study was conducted at a single center, which may limit the external validity of the findings. Multi-center studies are recommended to ensure that the results are applicable to a broader population.

Future research should continue exploring and validating effective self-management strategies for stress and sleep quality across various populations, considering the potential benefits these strategies can offer in improving personal well-being and reducing healthcare system burdens. Additionally, studies should consider expanding the sample size and incorporating comparative interventions to enhance the robustness of findings and allow for broader generalizations across different genders and types of interventions.

## 5 Conclusions

This randomized controlled study shows that the self-management of health sciences female students through GPR helps to reduce the state of stress and improve the quality of sleep, providing a useful and valuable tool for improve academic performance of their studies and future professional activity.

## Data availability statement

The raw data supporting the conclusions of this article will be made available by the authors, without undue reservation.

## Ethics statement

The studies involving humans were approved by Research ethics committee of Francisco de Vitoria University (UFV 18/2021). The studies were conducted in accordance with the local legislation and institutional requirements. The participants provided their written informed consent to participate in this study.

## Author contributions

MR-A: Conceptualization, Data curation, Project administration, Supervision, Validation, Writing – original draft, Writing – review & editing, Formal analysis, Funding acquisition. DV-D: Formal analysis, Investigation, Methodology, Software, Supervision, Visualization, Writing – original draft, Writing – review & editing. JG-H: Data curation, Resources, Writing – review & editing. AF-E: Data curation, Resources, Writing – review & editing. BM-H: Data curation, Resources, Writing – review & editing. NV: Data curation, Investigation, Methodology, Supervision, Validation, Visualization, Writing – review & editing.
